# Organic charge-modulated transistor for electrophysiological measurements of human-derived neurospheroids

**DOI:** 10.3389/fbioe.2025.1571011

**Published:** 2025-05-16

**Authors:** Fabio Terranova, Fabrizio Antonio Viola, Donatella Di Lisa, Paolo Massobrio, Sergio Martinoia, Annalisa Bonfiglio, Andrea Spanu

**Affiliations:** ^1^ Department of Informatics, Bioengineering, Robotics and System Engineering, University of Genova, Genova, Italy; ^2^ University School for Advanced Studies (IUSS), Pavia, Italy; ^3^ Department of Electrical and Electronic Engineering, University of Cagliari, Cagliari, Italy

**Keywords:** neurospheroids, OCMFET, hiPSC, bioelectronics, neural recording, organic transistors, 3D cell cultures

## Abstract

In this work, we present an alternative system to standard microelectrode arrays for monitoring the electrical activity of 3D cellular aggregates such as neurospheroids, which are known to better replicate the complex architecture and cellular interactions of native neural tissue than 2D cultures. The system is based on an ultra-sensitive organic sensor called Organic Charge-Modulated Field Effect Transistor (OCMFET) fabricated through low-resolution, low-cost fabrication techniques. This peculiar organic charge sensor offers interesting features like the absence of a reference electrode in the culture medium, a direct charge amplification, mechanical flexibility, and optical transparency. As a preliminary validation, the OCMFET system has been coupled to rtTA/Ngn2-positive human induced pluripotent stem cell (hiPSC)-derived neurospheroids and was able to reliably detect their spontaneous electrical activity exhibiting a high SNR. This preliminary validation lays the foundation for the development of simple, low-cost, and ultra-flexible organic transistor-based systems for high-performing, reliable interfacing with 3D neuronal structures.

## 1 Introduction

Investigating neural tissue function and dynamics is essential for understanding brain behavior and developing effective treatments for neurological disorders. Although traditional methodologies, such as standard planar cell cultures, have significantly advanced our understanding of mechanisms at the cellular and network levels, these two-dimensional (2D) models often fail to replicate the complex architecture and multicellular interactions characteristic of native neural tissue.

The emergence of three-dimensional (3D) neural cell culture models, particularly brain organoids and neurospheroids, has significantly advanced *in vitro* research (Zhuang et al., 2018). These models more accurately represent the *in vivo* environment of the neural tissue, recapitulating key characteristics, such as tissue architecture, cell organization, and both cell-cell and cell-matrix interactions. For example, cells within neurospheroids have been shown to exhibit enhanced expression of proteins that are particularly crucial for neural development and function, and generally display a more mature phenotype compared to their 2D counterparts (Simão et al., 2018; Camp et al., 2015; Jung et al., 2013).

The development of efficient methods for generating neurospheroids has further expanded their research applications. For instance, the hanging-drop technique enables the scaffold-free formation of uniform spheroidal aggregates without the need for bioreactors. This approach allows for the parallel culture of multiple neurospheroids within the same plate, reducing sample-to-sample variability and facilitating high-throughput studies (Durens et al., 2020). To effectively monitor cellular activity in these 3D cellular models, various technologies have been utilized and developed in the last years. For example, standard microelectrode arrays (MEAs), typically used for planar cultures, have been widely employed to record the electrical activity of brain organoids and neurospheroids (Muzzi et al., 2023; Jordan et al., 2024), while particular 3D MEAs have been specifically designed to interface with these complex 3D structures (Spanu et al., 2020; Yadav et al., 2023; Zips et al., 2023; Yang et al., 2024). However, MEAs passively record extracellular signals, requiring external amplification circuitry and the presence of a reference electrode, which limits system miniaturization.

Recently, organic field-effect transistors (OFETs) have emerged as a promising alternative for bioelectronic interfaces, both *in vivo* and *in vitro* (Spanu et al., 2021; Gu et al., 2019; Xie et al., 2020; Kyndiah et al., 2023). These devices exploit the unique properties of organic materials, such as flexibility, biocompatibility, and low-cost fabrication, and can be manufactured using low-temperature processes such as spin coating, inkjet printing, and screen printing (Forrest, 2004). However, conventional OFET-based biosensors integrate sensing and amplification within the same semiconductor layer, making them susceptible to environmental variations and reducing long-term stability. In this context, organic charge-modulated field-effect transistors (OCMFETs) offer distinct advantages over other MEAs and standard OFETs. Their operation relies on the modulation of the transistor channel conductivity induced by the presence of a charge on the surface of the sensing area, which can be read out as a variation of the threshold voltage of the device. Another key advantage of this organic transistor lies in its extended, floating-gate architecture and the presence of a control gate, which eliminates the need for a reference electrode in the solution, enabling further miniaturization and simplifying experimental setups (Spanu et al., 2015). In particular, OCMFETs are known to be very high-sensitive charge sensors, thanks to their double gate configuration, which provides an intrinsic signal amplification compared to standard OFETs. Additionally, unlike standard OFET-based interfaces, in the OCMFET architecture the sensing area and the organic semiconductor channel are physically separated, allowing for encapsulation that protects the organic semiconductor from degradation in humid biological environments – a crucial feature for long-term monitoring of cell cultures. Moreover, the sensing area can be specifically engineered and functionalized to detect other relevant parameters, such as metabolic cellular activity (Spanu et al., 2018). This feature is particularly convenient as it allows to develop multi-sensing platforms using the same transistor architecture (Spanu et al., 2022).

In this work, we demonstrate the capability of OCMFETs to successfully record the electrical activity of human induced pluripotent stem cell (hiPSC)-derived neurospheroids, confirming their potential for 3D *in vitro* applications. Furthermore, the ability to fabricate these devices on sub-micron plastic substrates enables the development of ultra-conformable systems (Viola et al., 2018), which can seamlessly integrate with complex 3D cellular structures while maintaining high signal fidelity. Combined with their low operating voltage, which reduces power consumption and minimizes undesired electrochemical reactions, OCMFET-based systems represent a powerful and scalable solution for next-generation bioelectronic interfaces.

## 2 Materials and methods

### 2.1 Device fabrication and characterization

**FIGURE 1:**
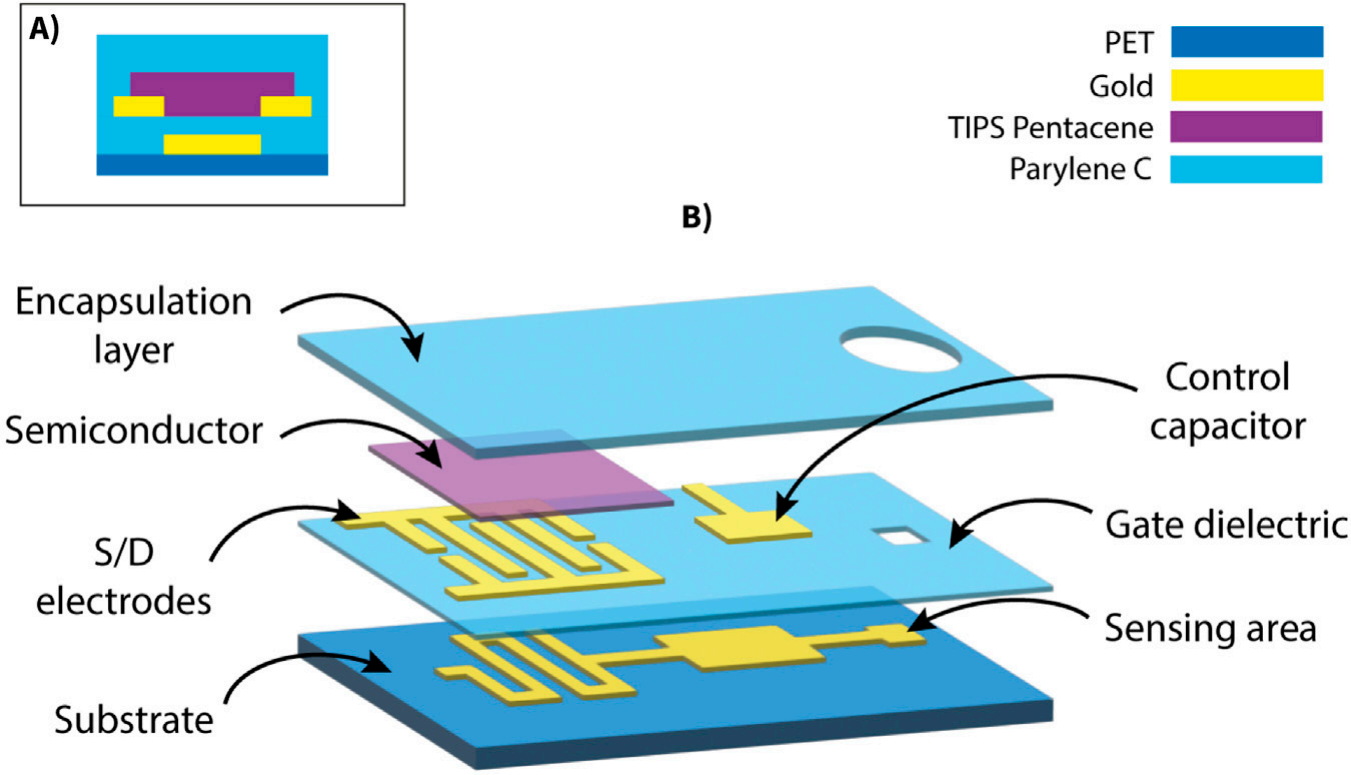
Structure of the OCMFET device. **(A)** Cross-sectional schematic showing the layered structure of the bottom-gate bottom-contact TIPS Pentacene transistor. **(B)** Exploded schematic view illustrating the multilayer architecture of the OCMFET.

The devices, each comprising two OCMFETs, were fabricated on polyethylene terephthalate (PET, 175 μm, Goodfellow) substrates (Figure 1). The first gold layer was thermally evaporated onto the substrate and patterned using a low-resolution photolithographic process. This metallic film served as the floating gate in the final sensors. A 200 nm Parylene C film was deposited onto the entire substrate through chemical vapor deposition (CVD) using the Labcoater 2 SCS PDS 2010 (Specialty Coating System). Subsequently, another gold layer was evaporated and patterned using a self-alignment process to form the interdigitated source and drain electrodes, the upper plate of the control gate capacitor, and the connection pads. A via was then created in the Parylene C layer by plasma oxygen etching (Tucano, Gambetti) to expose the sensing area (200 µm × 200 µm) of each OCMFET. A 2 µL droplet of 6,13-bis(triisopropylsilylethynyl)pentacene (TIPS pentacene) in anisole (1% w/v) was then drop-cast over the transistor channel, resulting in low-voltage OFETs with a channel width (W) of 25 mm and a channel length (L) of 40 µm ( W/L = 625). To enhance the stability of TIPS pentacene in humid environments (i.e., during incubation), the channel area of the devices was then covered with a thick (1.2 µm) layer of Parylene C. This step induces a slight shift of the threshold voltage, as already reported in the past (Lagomarsini et al., 2020), due to the fact that this layer embeds charge, and the presence of this static charge on top of the semiconductor layer influences the threshold voltage. After this last step, since no additional functionalization is added to the sensing area, the devices are able to reliably transduce only the faster cellular electrical activity, being basically immune to slow aspecific adsorption on the sensing surface (which can be readout as a slow drift and easily filtered out).

To evaluate the performance of the devices the charge carrier mobility (μ) and the threshold voltage $\left(V_{th}\right)$ of the transistors were extracted from the measured transfer characteristics. In the saturation region, the drain current $\left(I_{ds}\right)$ can be described by the following equation:

$$I_{ds}=\frac{1}{2}\mu C\frac{W}{L}{\left(V_{gs}-V_{th}\right)}^{2},$$

where $V_{gs}$ is the floating gate voltage, C is the gate dielectric capacitance per unit area, W is the channel width, and L is the channel length. By taking the square root of the absolute value of both sides, we obtain:

$$\sqrt{\left|I_{ds}\right|}=\sqrt{\frac{1}{2}\mu C\frac{W}{L}}|V_{gs}-V_{th}|.$$

Approximating the behavior of the OCMFET with this model, the square root of the absolute drain current can be fitted with a linear function against the gate voltage. The x-intercept of the fitted line, where $\sqrt{\left|I_{ds}\right|}=0$ , corresponds to the threshold voltage. The mobility value was estimated using the following relation:

$$\mu =\frac{m^{2}}{\frac{1}{2}C\frac{W}{L}},$$

where m is the slope of the fitted line, and the value of C was assumed to be 13.5 nF/cm^2^, as reported in a previous work for the same dielectric layer (Viola et al., 2018).

### 2.2 Neurospheroids generation and plating

The neurospheroids were derived from hiPSCs and prepared following established protocols (Muzzi et al., 2023). Specifically, this study employed a well-characterized rtTA/Ngn2-positive hiPSC line, which was derived from the fibroblasts of a healthy 30-year-old female. This hiPSC line, generated via episomal reprogramming, was obtained in frozen vials from the Coriell Institute for Medical Research (GM25256) and provided by Frega et al. The rtTA/Ngn2-positive lentiviral vectors were stably integrated into the hiPSC genome, enabling precise neuronal differentiation into early-stage excitatory cortical layer 2/3 neurons (iNeurons) through doxycycline treatment for 3 days. Maintenance of the cell line was performed following previously established protocols (Frega et al., 2017; Muzzi et al., 2021). The neurospheroids were composed of 50,000 iNeurons and astrocytes in a 1:1 ratio. The *hanging-drop* method was employed to create spherical aggregates of cells, aiming to generate neurospheroids in a scaffold-free culture system.

A 5 cm Petri dish was used as a “moisture chamber,” with the bottom partially filled with Dulbecco’s Phosphate-Buffered Saline (DPBS). The inverted lid of the dish was used to hold 15 µL drops of neurobasal medium, into which 15 µL drops of a mixed-cell solution (neurons and astrocytes in a 1:1 ratio, at a density of 50,000 cells per drop) were added. The dish was carefully reassembled and incubated at 37°C with 5.5% CO_2_. The neurobasal medium was based on Neurobasal (Thermo Fisher Scientific), supplemented with 2% B-27 supplement (Thermo Fisher Scientific), 1% GlutaMAX (Thermo Fisher Scientific), 0.1% human BDNF (Thermo Fisher Scientific), 0.1% human NT-3 (Thermo Fisher Scientific), 4 μg/mL doxycycline (Sigma Aldrich), and 1% penicillin/streptomycin (Sigma Aldrich).

On day *in vitro* (DIV) 1, Cytosine β -D-arabinofuranoside (Ara-C, Sigma Aldrich), a mitotic inhibitor, was introduced to inhibit the proliferation of non-neuronal cells. To maintain optimal conditions for cell survival and development, medium changes (50% volume) were performed three times per week. By DIV 10, the neurospheroids were transferred to 24-well plates pre-coated with a 1% w/v alginate solution. This coating prevented cell attachment to the plate surface, preserving the spheroidal structure. From this stage onward, the culture medium was supplemented with 2.5% fetal bovine serum (FBS) to enhance cellular growth and differentiation. Medium changes continued three times a week, with 50% of the medium replaced each time to provide a consistent nutrient supply and prevent the accumulation of waste.

At DIV 21, neurospheroids were transferred onto OCMFET devices using a pipette. Before plating, the devices were prepared by sequential cleaning with deionized water, sterilizing with ethanol, and coating with cell-adhesion promoters (poly-L-ornithine and laminin). Following deposition, the neurospheroids were incubated at 37°C with 5.5% CO_2_. To maintain neurospheroid viability, the culture medium was partially refreshed, replacing 50% of the volume three times per week.

### 2.3 Electrophysiological recording setup

The electrical activity of the neurospheroids coupled with the OCMFET devices was recorded using a custom data acquisition system developed in collaboration with Elbatech (https://www.elbatech.it/). This system simultaneously recorded the drain current $\left(I_{ds}\right)$ of two channels at a sampling rate of 22.7 kHz and, via a feedback control loop, automatically adjusted the drain potential $\left(V_{ds}\right)$ of the OCMFETs to maintain a constant baseline, thus removing the typical drift component of OFETs. Each device contained two individual transistors, with one of the two OCMFETs coupled with a single neurospheroid, while the second was used as a control. The neurospheroids were maintained in incubation for 7 days prior to the recording sessions, with the culture media refreshed three times per week. All measurements were conducted at 37°C and 5.5% CO_2_ to ensure optimal conditions for the 3D neural cultures. Recording sessions, each lasting 10–15 min, were performed from DIV 28 onwards.

### 2.4 Immunofluorescence

Following electrophysiological recordings, the samples were fixed at DIV 35 for fluorescence imaging. Fixation was carried out using a 4% paraformaldehyde (PFA, Sigma-Aldrich) solution for 20 min at room temperature, followed by three washes with phosphate-buffered saline (PBS, Sigma-Aldrich). Samples were permeabilized using 0.2% Triton X-100 (Thermo Fisher Scientific) for 15 min and subsequently blocked with a Blocking Buffer Solution (BBS) composed of 0.5% fetal bovine serum (FBS, Sigma-Aldrich) and 0.3% bovine serum albumin (BSA, Sigma-Aldrich) in PBS for 45 min at room temperature. Primary antibodies, GFAP (1:500, glial fibrillary acidic protein, Cat. 173 002 and 173 01, Synaptic System) and MAP-2 (1:500, dendritic microtubule-associated protein, Cat. 188 002 and 188,011, Synaptic System), were applied to label glial and neuronal cells, respectively. Secondary antibodies included Alexa Fluor 488 (1:700, Thermo Fisher Scientific) and Alexa Fluor 546 (1:1,000, Invitrogen), along with goat anti-mouse or goat anti-rabbit antibodies. Confocal imaging was conducted on a Leica STELLARIS 8 Falcon τ -STED inverted confocal/STED microscope (Leica Microsystems, Mannheim, Germany). Data analysis was performed using LASX V2.0 software (Leica Microsystems Srl, Italy) for primary image acquisition and processing. In addition, the Fiji image processing package was used to analyze the acquired data.

### 2.5 Data analysis

The recorded signals were processed and analyzed using Python. The pre-processing phase consisted of filtering the raw data with a second-order zero-phase Butterworth filter to retain frequencies within the range of 10 Hz to 5 kHz, effectively reducing noise and isolating relevant signal components. Spike detection was performed using a threshold-based approach, identifying events as negative peaks exceeding 5 standard deviations below the baseline signal. For each identified negative peak, a corresponding positive peak was detected by locating the maximum positive deflection within a 150 m window following the negative peak. Three features were extracted from each detected event: duration, calculated as the time difference between the positive and negative peak positions; negative peak amplitude; and amplitude (or peak-to-peak amplitude), measured as the current difference between the positive and negative peaks;

To analyze and categorize detected events, principal component analysis (PCA) was performed on the three extracted features, reducing the dimensionality of the data to two principal components while preserving the variance in the original dataset. The resulting events in the two-dimensional representation were then clustered using the k-means algorithm (k=3) to identify distinct event populations.

The recorded signals were also analyzed in the frequency domain to compare the spectral characteristics between the baseline (in the absence of neurospheroids) and recordings containing detected events. The power spectral density (PSD) was calculated for both conditions using Welch’s method. The signal-to-noise ratio (SNR) of the recorded signals was determined using the formula:

$$\text{SNR}={\left(\frac{{\text{RMS}}_{\text{signal}}}{{\text{RMS}}_{\text{noise}}}\right)}^{2}$$

where ${\text{RMS}}_{\text{signal}}$ represents the root mean square (RMS) of the time series of detected events, and ${\text{RMS}}_{\text{noise}}$ corresponds to the RMS of the baseline noise recorded from the same channel.

## 3 Results

### 3.1 OCMFET device characterization

**FIGURE 2:**
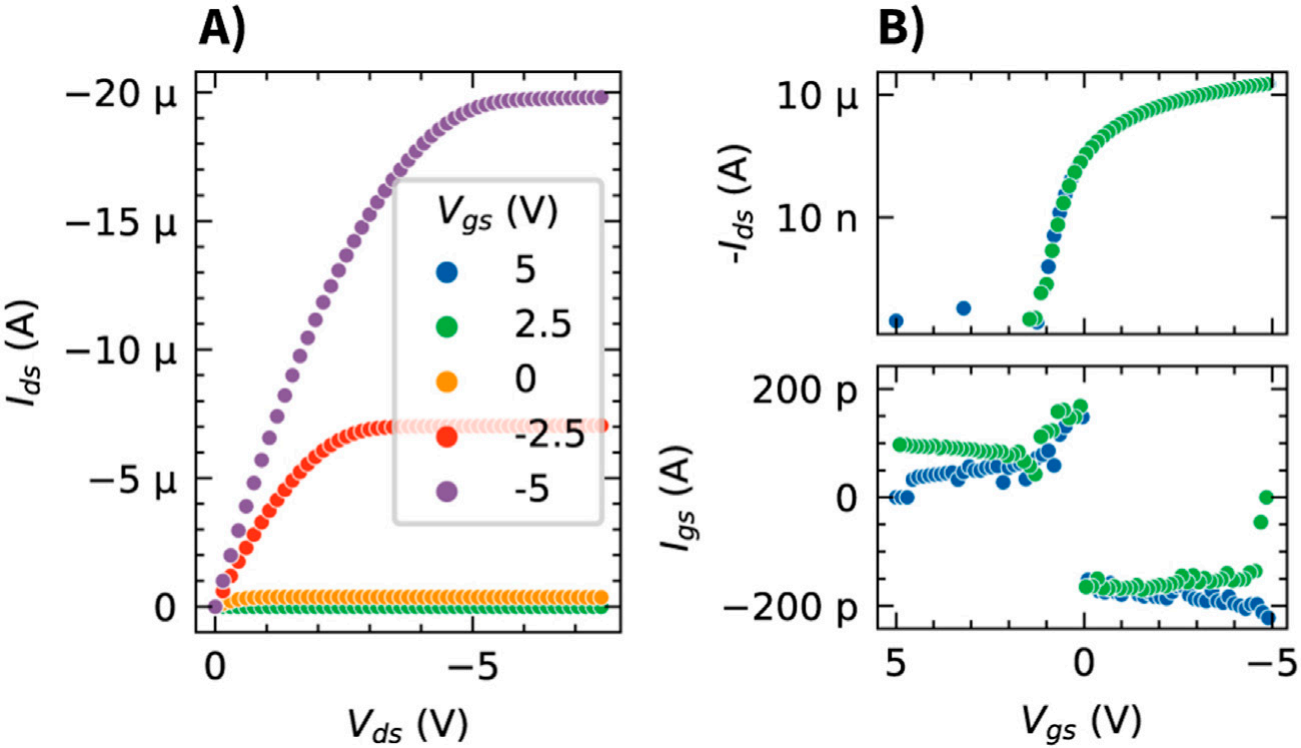
Electrical characterization of the TIPS pentacene based OCMFET. **(A)** Output characteristics ( $I_{ds}$ - $V_{ds}$ ) measured at different gate-source voltages ( $V_{gs}$ ). **(B)** Transfer characteristics ( $I_{ds}$ - $V_{gs}$ and $I_{gs}$ - $V_{gs}$ ) in both forward (blue) and backward (green) voltage sweeps, measured with $V_{ds}$ = −7.5 V.

**FIGURE 3:**
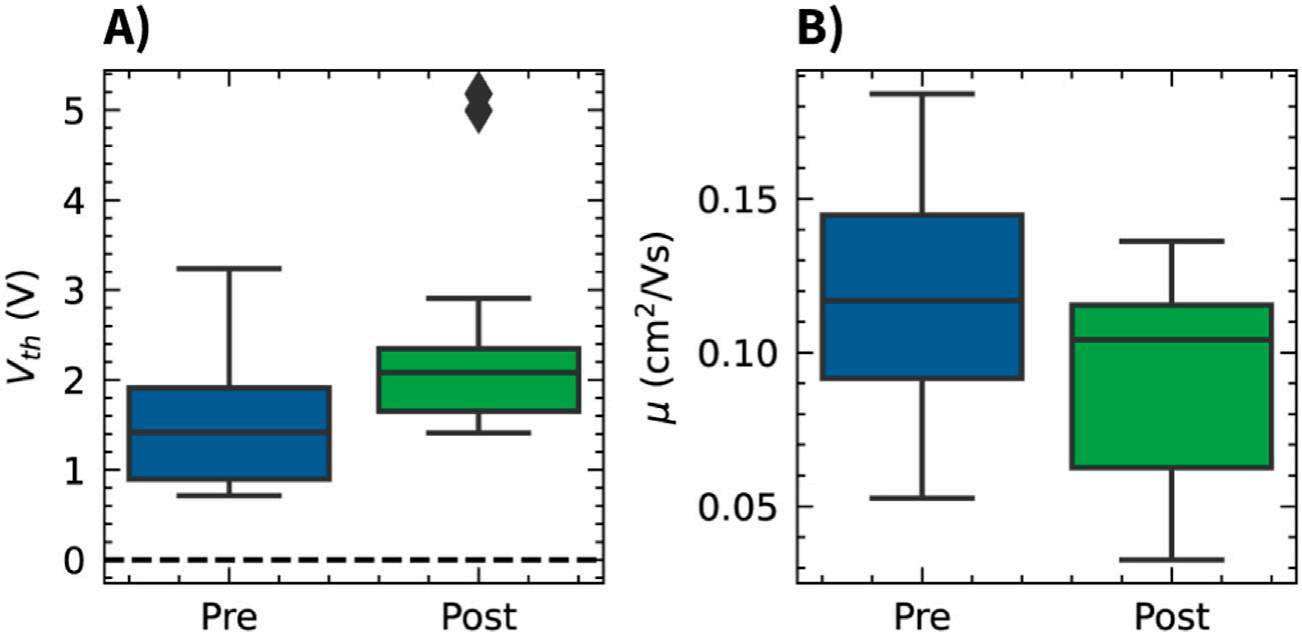
Effect of the Parylene C encapsulation on OCMFET electrical parameters. Box plots comparing the threshold voltage **(A)** and charge carrier mobility **(B)** distributions before (pre) and after (post) the encapsulation process.

The fabricated OCMFET devices were characterized prior to being used in the electrophysiology experiments. The transfer curves (Figure 2) demonstrate the low-voltage operation of the TIPS pentacene OFET, with a threshold voltage of  (1.4±0.7) V and charge carrier mobility of (0.117±0.039) cm^2^/(Vs.) (mean ± SD, n=12 transistors). Importantly, the OCMFET performance was maintained after the encapsulation step (with a slight shift of $V_{th}$ towards more positive values and a slight decrease in charge carrier mobility, as shown in Figure 3), which represents a very important aspect when long-term monitoring applications are considered.

### 3.2 Neurospheroid characterization

**FIGURE 4:**
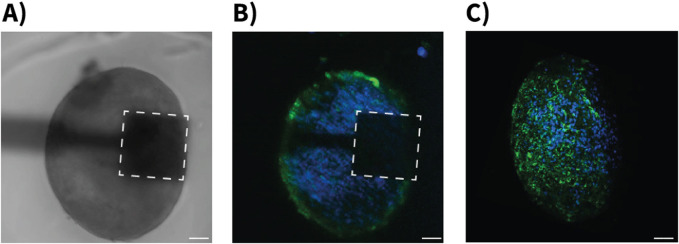
Morphological characterization of hiPSC-derived neurospheroids on OCMFET devices. **(A)** Phase-contrast microscopy image showing a neurospheroid positioned on the sensing area (dashed square) of a OCMFET (scale bar: 100 µm). **(B)** Confocal fluorescence microscopy image of the neurospheroid on device, showing the distribution of neurons (blue, MAP-2) and glial cells (green, GFAP) (scale bar: 100 µm). **(C)** Projection of confocal z-stack of a neurospheroid after device detachment, showing the three-dimensional cellular organization with neuronal (blue) and glial (green) components throughout the spheroid structure (scale bar: 100 µm).

The neurospheroids used in this study were characterized using both phase-contrast and fluorescence microscopy. Phase-contrast imaging revealed compact, spherical structures with well-defined boundaries positioned on the sensing areas of the devices (Figure 4A). To confirm the cellular composition of the neurospheroids, immunofluorescence staining was performed using antibodies against MAP-2 (Microtubule-Associated Protein 2) to label neurons and GFAP (Glial Fibrillary Acid Protein) to label astrocytes. These were visualized using Alexa Fluor 488 (green) and Alexa Fluor 546 (red) secondary antibodies, respectively. This analysis confirmed the presence of both neurons and astrocytes within the neurospheoroid structure (Figure 4B). Confocal z-stack images revealed a three-dimensional cellular organization that is consistent with previous reports of hiPSC-derived neurospheroids, with astrocytes predominantly distributed along the periphery of the spheroids (Figure 4C).

### 3.3 Activity features and spectral analysis

**FIGURE 5:**
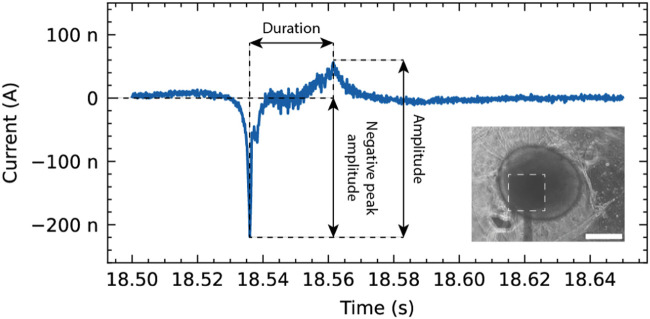
Representative event recorded from a neurospheroid using the OCMFET device. The trace shows the temporal evolution of the recorded signal, highlighting key features such as duration, amplitude, and negative peak amplitude. Inset: Phase-contrast microscopy image showing the neurospheroid positioned on the sensing area (dashed square) of the device (scale bar: 200 µm).

**FIGURE 6:**
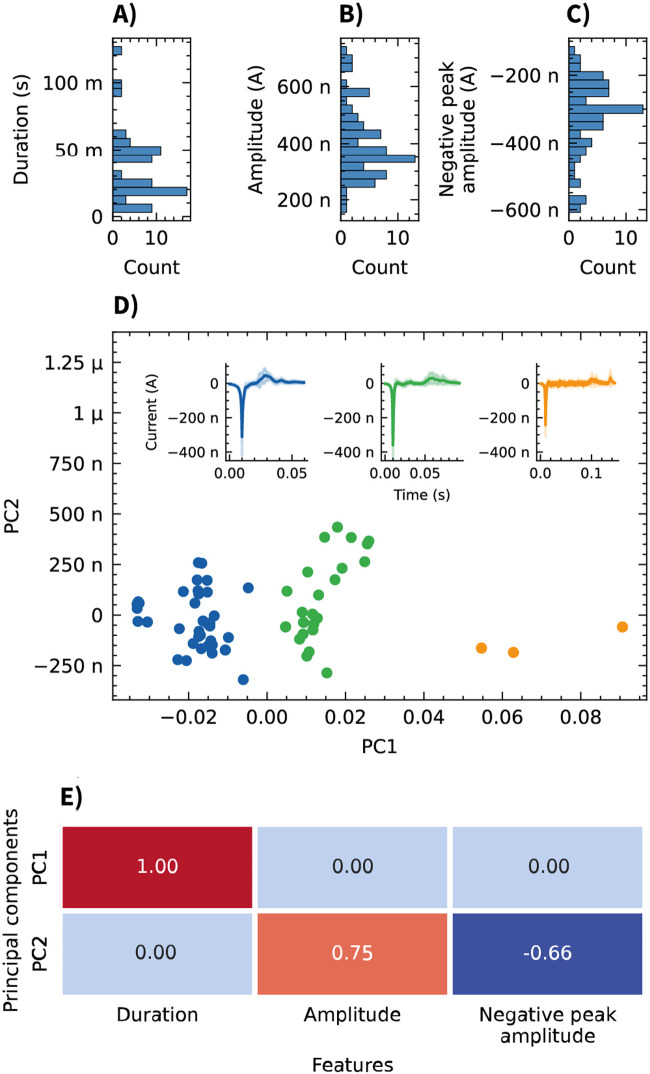
Analysis of event features recorded from neurospheroids using OCMFETs. **(A–C)** Histograms showing the distribution of event features: duration **(A)**, amplitude **(B)**, and negative peak amplitude **(C)**. **(D)** Principal component analysis (PCA) of the three features reveals three clusters (blue, green, and orange). Insets show the average event waveforms (solid lines) and standard deviations (shaded areas) for each cluster. **(E)** Heatmap of the PCA loadings showing the contributions of each feature (duration, amplitude, and negative peak amplitude) to PC1 and PC2.

Analysis of the recorded signals revealed distinct spike-like events in the OCMFET recordings. Using a threshold set at 5 standard deviations from the baseline, consistent bi-phasic spikes characterized by a negative deflection followed by a positive peak were detected (Figure 5). The distribution of the extracted features (event amplitude, duration, and negative peak magnitude) across all recorded events is shown in Figures 6A–C. To analyze the heterogeneity of the detected events and address potential redundancy in the extracted features, principal component analysis (PCA) was applied. Particularly amplitude and negative peak amplitude were hypothesized to be correlated. The application of PCA revealed three distinct classes visible in the scatter plot of events projected onto this new feature space. Another motivation for this analysis was to facilitate the visualization of the average event waveform. Given the apparent variability in the shapes of the events, averaging all detected events together would have provided a non-representative picture of the diverse shapes of the detected events. K-means clustering identified three representative patterns within the recorded events (Figure 6D). These clusters might represent different cellular populations or varying forms of network activity within the 3D neural tissue. The contributions of the extracted features (duration, amplitude, and negative peak amplitude) to the two principal components (PC1 and PC2), as depicted in Figure 6E, show PC1 exclusively driven by the event duration with a loading of 1.00, while PC2 exhibits a positive loading of 0.75 for amplitude and a negative loading of −0.66 for negative peak amplitude. This confirms the initial hypothesis that amplitude and negative peak amplitude contributed to a common source of variance, captured by PC2.

**FIGURE 7:**
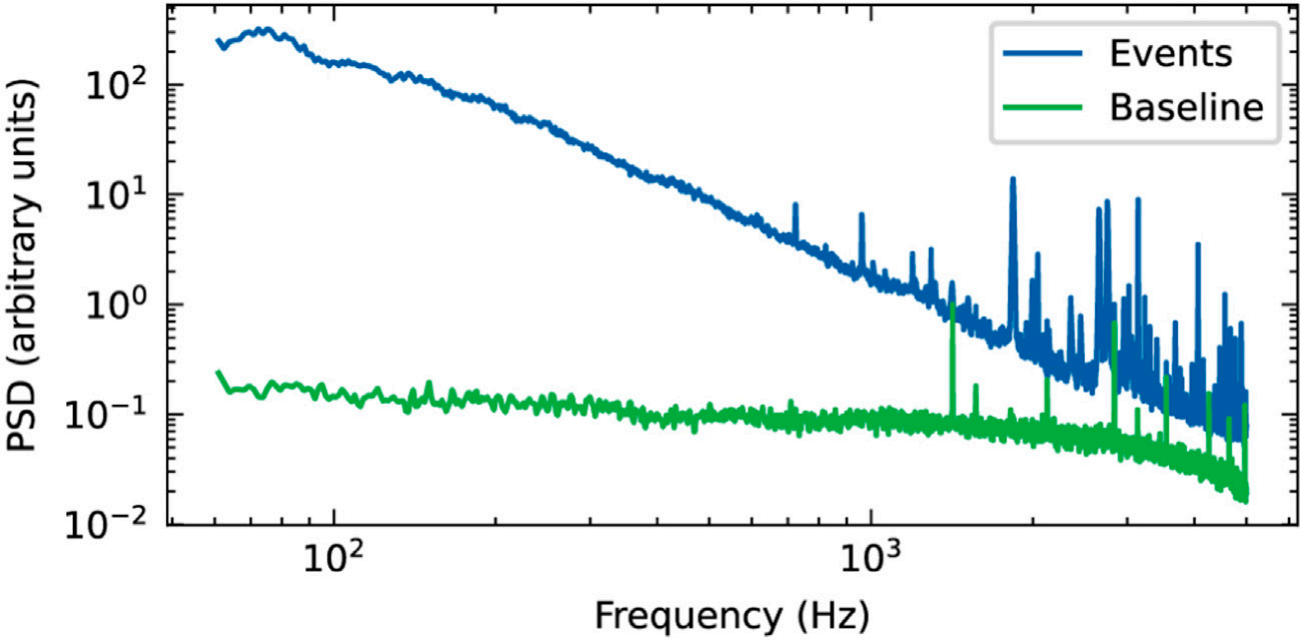
Power spectral density (PSD) analysis of OCMFET recordings from neurospheroids. Comparison of frequency components with (events, blue) and without (baseline, green) neurospheroid.

**FIGURE 8:**
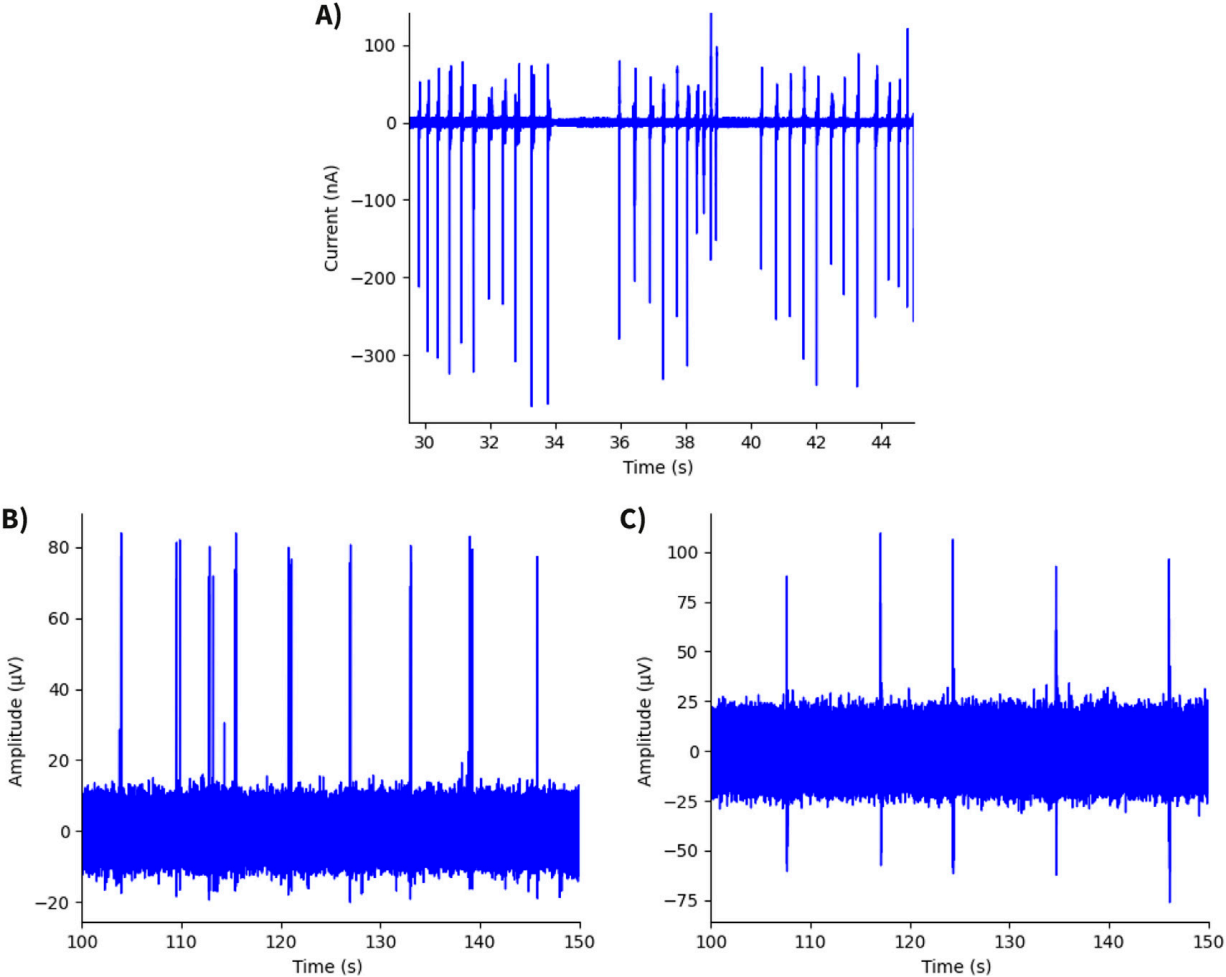
Time traces of neurospheroid recordings. **(A)** Recording from hiPSC-derived neurospheroid using an OCMFET device, showing a SNR of 589. **(B)** Recording from hiPSC-derived neurospheroid using an MEA60 system (Multichannel Systems), with a SNR of 45. **(C)** Recording from rat cortical neurospheroid using an MEA60 system, exhibiting the lowest value of SNR (Yang et al., 2024).

Power spectral density (PSD) analysis showed a clear difference between recordings with and without neurospheroids present on the sensing area of the OCMFET, with significantly higher power observed when cells were present (Figure 7). The PSD of neurospheroids recordings exceeded the baseline by approximately two orders of magnitude at low frequencies ( < 1 kHz) and showed distinct peaks at higher frequencies, exhibiting a high signal-to-noise ratio (SNR = 589) in comparison with both hiPSC-derived and rat cortical neurospheroid activity recorded with traditional MEAs (Figure 8).

## 4 Discussion

In this work, we have tested organic charge-modulated field-effect transistors (OCMFETs) for recording the spontaneous, synchronized electrical activity of human induced pluripotent stem cell (hiPSC)-derived neurospheroids. The proposed devices show good electrical performance at low operating voltages, an important aspect when dealing with in-liquid measurements in that it helps to slow down the degradation of the sensing area material, it reduces unwanted perturbation of cells activity, and it allows for ultra-low power consumption.

The recorded events captured the spontaneous electrical activity of the hiPSC-derived neurospheroids, demonstrating the ability of OCMFET devices to transduce the electrophysiological responses of these 3D cell cultures. A key next step in the validation of this platform involves testing these devices across different neural models and stimulation protocols, including pharmacological modulation. In parallel, scaling down the dimensions of the sensitive areas will enable higher density mapping of electrical activity within tissues.

Although preliminary, this evaluation highlighted excellent performance in terms of signal transduction, thus laying the groundwork for future iterations of the OCMFET technology. In particular, this solution, thanks to the versatility of the transduction principle and the possibility of fabricating these devices on ultra-flexible, possibly conformable substrates, will drive the development of a new generation of high-performing devices for recording the electrical activity of 3D complex cellular aggregates *in vitro*, thus enabling innovative applications in precision medicine, drug discovery, and high-throughput screening.

## Data Availability

The raw data supporting the conclusions of this article will be made available by the authors, without undue reservation.

## References

[B1] Camp J. G. Badsha F. Florio M. Kanton S. Gerber T. Wilsch-Bräuninger M. (2015). Human cerebral organoids recapitulate gene expression programs of fetal neocortex development. Proc. Natl. Acad. Sci. 112, 15672–15677. 10.1073/pnas.1520760112 26644564 PMC4697386

[B2] Durens M. Nestor J. Williams M. Herold K. Niescier R. F. Lunden J. W. (2020). High-throughput screening of human induced pluripotent stem cell-derived brain organoids. J. Neurosci. Methods 335, 108627. 10.1016/j.jneumeth.2020.108627 32032714

[B3] Forrest S. R. (2004). The path to ubiquitous and low-cost organic electronic appliances on plastic. Nature 428, 911–918. 10.1038/nature02498 15118718

[B4] Frega M. Van Gestel S. H. C. Linda K. Van Der Raadt J. Keller J. Van Rhijn J. R. (2017). Rapid neuronal differentiation of induced pluripotent stem cells for measuring network activity on micro-electrode arrays. J. Vis. Exp., 54900. 10.3791/54900 28117798 PMC5407693

[B5] Gu X. Yeung S. Y. Chadda A. Poon E. N. Y. Boheler K. R. Hsing I. M. (2019). Organic electrochemical transistor arrays for *in vitro* electrophysiology monitoring of 2D and 3D cardiac tissues. Adv. Biosyst. 3, 1800248. 10.1002/adbi.201800248 32627368

[B6] Jordan F. D. Kutter M. Comby J. M. Brozzi F. Kurtys E. (2024). Open and remotely accessible Neuroplatform for research in wetware computing. Front. Artif. Intell. 7, 1376042. 10.3389/frai.2024.1376042 38756757 PMC11097343

[B7] Jung G. S. Lee K. M. Park J. K. Choi S. K. Jeon W. B. (2013). Morphogenetic and neuronal characterization of human neuroblastoma multicellular spheroids cultured under undifferentiated and all-trans-retinoic acid-differentiated conditions. BMB Rep. 46, 276–281. 10.5483/BMBRep.2013.46.5.196 23710639 PMC4133894

[B8] Kyndiah A. Dipalo M. Molazemhosseini A. Viola F. A. Modena F. Iachetta G. (2023). Direct recording of action potentials of cardiomyocytes through solution processed planar electrolyte-gated field-effect transistors. Sensors Actuators B Chem. 393, 134227. 10.1016/j.snb.2023.134227

[B9] Lagomarsini C. Jean-Mistral C. Kachroudi A. Monfray S. Sylvestre A. (2020). Outstanding performance of parylene polymers as electrets for energy harvesting and high-temperature applications. J. Appl. Polym. Sci. 137, 48790. 10.1002/app.48790

[B10] Muzzi L. Di Lisa D. Arnaldi P. Aprile D. Pastorino L. Martinoia S. (2021). Rapid generation of functional engineered 3D human neuronal assemblies: network dynamics evaluated by micro-electrodes arrays. J. Neural Eng. 18, 066030. 10.1088/1741-2552/ac3e02 34844234

[B11] Muzzi L. Di Lisa D. Falappa M. Pepe S. Maccione A. Pastorino L. (2023). Human-derived cortical neurospheroids coupled to passive, high-density and 3D MEAs: a valid platform for functional tests. Bioengineering 10, 449. 10.3390/bioengineering10040449 37106636 PMC10136157

[B12] Simão D. Silva M. M. Terrasso A. P. Arez F. Sousa M. F. Q. Mehrjardi N. Z. (2018). Recapitulation of human neural microenvironment signatures in iPSC-derived NPC 3D differentiation. Stem Cell Rep. 11, 552–564. 10.1016/j.stemcr.2018.06.020 PMC6094163 30057262

[B13] Spanu A. Colistra N. Farisello P. Friz A. Arellano N. Rettner C. T. (2020). A three-dimensional micro-electrode array for *in-vitro* neuronal interfacing. J. Neural Eng. 17, 036033. 10.1088/1741-2552/ab9844 32480394

[B14] Spanu A. Lai S. Cosseddu P. Tedesco M. Martinoia S. Bonfiglio A. (2015). An organic transistor-based system for reference-less electrophysiological monitoring of excitable cells. Sci. Rep. 5, 8807. 10.1038/srep08807 25744085 PMC4351515

[B15] Spanu A. Martines L. Bonfiglio A. (2021). Interfacing cells with organic transistors: a review of *in vitro* and *in vivo* applications. Lab a Chip 21, 795–820. 10.1039/D0LC01007C 33565540

[B16] Spanu A. Martines L. Tedesco M. Martinoia S. Bonfiglio A. (2022). Simultaneous recording of electrical and metabolic activity of cardiac cells *in vitro* using an organic charge modulated field effect transistor array. Front. Bioeng. Biotechnol. 10, 945575. 10.3389/fbioe.2022.945575 35992349 PMC9385991

[B17] Spanu A. Tedesco M. T. Martines L. Martinoia S. Bonfiglio A. (2018). An organic neurophysiological tool for neuronal metabolic activity monitoring. Apl. Bioeng. 2, 046105. 10.1063/1.5050170 31069327 PMC6481818

[B18] Viola F. A. Spanu A. Ricci P. C. Bonfiglio A. Cosseddu P. (2018). Ultrathin, flexible and multimodal tactile sensors based on organic field-effect transistors. Sci. Rep. 8, 8073. 10.1038/s41598-018-26263-1 29795264 PMC5966445

[B19] Xie K. Wang N. Lin X. Wang Z. Zhao X. Fang P. (2020). Organic electrochemical transistor arrays for real-time mapping of evoked neurotransmitter release *in vivo* . eLife 9, e50345. 10.7554/eLife.50345 32043970 PMC7075691

[B20] Yadav N. Lisa D. D. Giacomozzi F. Cian A. Giubertoni D. Martinoia S. (2023). Development of multi-depth probing 3D microelectrode array to record electrophysiological activity within neural cultures. J. Micromechanics Microengineering 33, 115002. 10.1088/1361-6439/acf940

[B21] Yang X. Forró C. Li T. L. Miura Y. Zaluska T. J. Tsai C. T. (2024). Kirigami electronics for long-term electrophysiological recording of human neural organoids and assembloids. Nat. Biotechnol. 42, 1836–1843. 10.1038/s41587-023-02081-3 38253880 PMC11260907

[B22] Zhuang P. Sun A. X. An J. Chua C. K. Chew S. Y. (2018). 3D neural tissue models: from spheroids to bioprinting. Biomaterials 154, 113–133. 10.1016/j.biomaterials.2017.10.002 29120815

[B23] Zips S. Huang B. Hotte S. Hiendlmeier L. Wang C. Rajamani K. (2023). Aerosol jet-printed high-aspect ratio micro-needle electrode arrays applied for human cerebral organoids and 3D neurospheroid networks. ACS Appl. Mater. and Interfaces 15, 35950–35961. 10.1021/acsami.3c06210 37469180

